# *Toxoplasma gondii* IgG associations with sleep-wake problems, sleep duration and timing

**DOI:** 10.1515/pteridines-2019-0001

**Published:** 2019-02-19

**Authors:** Celine C. Corona, Man Zhang, Abhishek Wadhawan, Melanie L. Daue, Maureen W. Groer, Aline Dagdag, Christopher A. Lowry, Andrew J. Hoisington, Kathleen A. Ryan, John W. Stiller, Dietmar Fuchs, Braxton D. Mitchell, Teodor T. Postolache

**Affiliations:** Mood and Anxiety Program, Department of Psychiatry, University of Maryland School of Medicine, Baltimore, MD 21201, USA; Division of Endocrinology, Diabetes and Nutrition, Department of Medicine, University of Maryland School of Medicine, Baltimers, MD 21201, USA; Mood and Anxiety Program, Department of Psychiatry, University of Maryland School of Medicine, Baltimore, MD 21201, USA, Saint Elizabeths Hospital, Psychiatry Residency Program, Washington, DC 20032, USA; Division of Endocrinology, Diabetes and Nutrition, Department of Medicine, University of Maryland School of Medicine, Baltimers, MD 21201, USA, Program for Personalized and Genomic Medicine, University of Maryland School of Medicine, Baltimore, MD 21201, USA, Geriatrics Research and Education Clinical Center, Veteran Affairs Medical Center, Baltimore, MD 21201, USA; College of Nursing, University of South Florida College of Nursing, Tampa, FL 33612, USA; Mood and Anxiety Program, Department of Psychiatry, University of Maryland School of Medicine, Baltimore, MD 21201, USA, Psychiatry Adult Inpatient & Behavioral Health, University of Maryland Medical Center, Baltimore, MD 21201, USA; Department of Integrative Physiology and Center for Neuroscience, University of Colorado Boulder, Boulder, CO 80309, USA; Department of Physical Medicine and Rehabilitation and Center for Neuroscience, University of Colorado Anschutz Medical Campus, Aurora, CO80045, USA, Rocky Mountain Mental Illness Research Education and Clinical Center (MIRECC), Veterans Integrated Service Network (VISN) 19, Military and Veteran. Microbiome: Consortium for Research and Education (MVM-CoRE), Aurora, CO80 045, USA; Rocky Mountain Mental Illness Research Education and Clinical Center (MIRECC), Veterans Integrated Service Network (VISN) 19, Military and Veteran. Microbiome: Consortium for Research and Education (MVM-CoRE), Aurora, CO80 045, USA, Department of Systems Engineering and Management, Air Force Institute of Technology, Wright-Patterson AFB, OH 45433, USA; Division of Endocrinology, Diabetes and Nutrition, Department of Medicine, University of Maryland School of Medicine, Baltimers, Md 21201, USA, Program for Personalized and Genomic Medicine, University of Maryland School of Medicine, Baltimore, MD 21201, USA; Mood and Anxiety Program, Department of Psychiatry, University of Maryland School of Medicine, Baltimore, MD 21201, USA, Saint Elizabeths Hospital, Department of Neurology, Washington DC 20032, USA; Maryland State Athletic Commission, Baltimore, MD 21202, USA; Division of Biological Chemistry, Biocenter, Innsbruck Medical University, Innsbruck, Austria; Division of Endocrinology, Diabetes and Nutrition, Department of Medicine, University of Maryland School of Medicine, Baltimers, MD 21201, USA, Program for Personalized and Genomic Medicine, University of Maryland School of Medicine, Baltimore, MD 21201, USA, Geriatrics Research and Education Clinical Center, Veteran Affairs Medical Center, Baltimore, MD 21201, USA

**Keywords:** IgG, mid-sleep time, sleep duration, sleep timing, *Toxoplasma gondii*

## Abstract

**Background:**

Evidence links *Toxoplasmagondii* (*T. gondii*), a neurotropic parasite, with schizophrenia, mood disorders and suicidal behavior, all of which are associated and exacerbated by disrupted sleep. Moreover, low-grade immune activation and dopaminergic overstimulation, which are consequences of *T. gondii* infection, could alter sleep patterns and duration.

**Methods:**

Sleep data on 833 Amish participants [mean age (SD) = 44.28 (16.99) years; 59.06% women] were obtained via self-reported questionnaires that assessed sleep problems, duration and timing. *T. gondii* IgG was measured with ELISA. Data were analyzed using multivariable logistic regressions and linear mixed models, with adjustment for age, sex and family structure.

**Results:**

*T. gondii* seropositives reported less sleep problems (*p* < 0.005) and less daytime problems due to poor sleep (*p* < 0.005). Higher *T. gondii* titers were associated with longer sleep duration (*p* < 0.05), earlier bedtime (*p*< 0.005) earlier mid-sleep time (*p* < 0.05).

**Conclusions:**

It seems unlikely that sleep mediates the previously reported associations between *T. gondii* and mental illness. Future longitudinal studies with objective measures are necessary to replicate our findings.

## Introduction

During infection, sleep and the immune system interact to improve the fitness of the host by better allocation of energy to critical physiological functions implicated in controlling the infection [1]. While the majority of studies focus on viral and bacterial infections, sleep is strongly altered in parasitic infections too. For instance, in the human African trypanosomiasis (sleeping sickness), there is a complete loss of circadian rhythm with narcolepticlike fits and decreased total hours of wakefulness [2]. Moreover, changes in sleep duration have been associated with parasitic infection in 12 mammalian species [3]. It has been suggested that sleep “fuels” the immune system to increase resistance to parasites [4]. Levels of cytokines such as interleukin (IL)-1β, IL-10, IL-12 and tumor necrosis factor (TNF) are at their highest during sleep, regardless of circadian rhythms [1]. Proinflammatory cytokine activity increases when a host is faced with an immune challenge, which leads to increased duration of non-rapid eye movement (NREM) sleep [4]. White blood cell counts have also been associated with the total sleep time in 26 mammalian species [3].

*Toxoplasma gondii* (*T. gondii*) is a protozoan parasite estimated to have infected 30% of the world’s population [5]. Among Americans, the prevalence of *T. gondii* seropositivity is approximately 13.2% [6]. It has the ability to invade numerous warm-blooded animals as intermediate hosts and members of the feline family, who are its definitive hosts [7]. Within the cat, *T. gondii* reproduces sexually and oocysts are formed and excreted into the environment where they are ingested by rodents and other intermediate hosts, including humans, through contamination of food and water [8]. Consuming contaminated undercooked meat also infects humans [9-13]. The environment, the host’s genetic framework, the specific strain of *T. gondii*, the parasitic stage and the route of contamination during inoculation, all contribute to the severity of *T. gondii* infection [14]. Many parasites [15], including *T. gondii* [16, 17], have the capability to change the behavior of their hosts to enhance the completion of their life cycle. The immune system of an immunocompetent host with chronic *T. gondii* infection contains the parasite to its slow-growing forms inside tissue cysts [18-20]. There has been growing evidence that suggests increased rates of *T. gondii* infection in individuals with mental illness, in particular schizophrenia, including new-onset schizophrenia [21, 22]. Sutterland et al. (2015) performed a meta-analysis and uncovered significant associations of *T. gondii* infection with schizophrenia, bipolar disorder and obsessive-compulsive disorder [23]. Recently, we reported that *T. gondii* IgG serointensity was positively associated with cardinal symptoms of depression in the Old Order Amish [24]. Links have also been identified between *T. gondii* IgG seropositivity or serointensity and suicidal behavior [25-29] and with an increased risk of traffic accidents [30-32].

Disturbances in sleep are more common and severe in those suffering from psychiatric conditions [33, 34], including schizophrenia [35-39], depression [40], bipolar disorder [41] and suicidal behavior [42-44], as well as car accidents [45], all of which, as stated above, have also been previously associated with *T. gondii* infection. We thus reasoned that sleep impairment might mediate, at least in part, the link between *T. gondii* infection and psychiatric disorders and behavioral dysregulation. Recently, we reported in a sample of Old Order Amish, no associations of *T. gondii* IgG seropositivity and serointensity with bedtime difficulties or daytime sleepiness [46].

Delayed sleep phase is associated with mood disturbances, such as unipolar depression and bipolar disorder [47]. Delayed timing and duration of sleep have been reported in bipolar depression [48, 49]. Moreover, severity of depressive symptoms is increased in those with delayed timing of sleep [50]. Sleep midpoint has also been reported to be delayed in those suffering from depression [51]. Given these data, we expected that later bedtime, mid-sleep time and wake-up time would be positively associated with *T. gondii* IgG serointensity or seropositivity.

Given that low-grade immune activation is known to alter sleep [52], together with the production by *T. gondii* of dopamine [53, 54], a wakefulness-promoting neurotransmitter, we hypothesized that *T. gondii* would be associated with changes in the duration of sleep, delayed timing of sleep, problems maintaining sleep and increased daytime problems due to poor sleep.

## Methods

### Study population

Data were drawn from the Amish Wellness Study, which began in 2010 as part of a cardio-metabolic screening program for the Amish community in Lancaster County, PA, USA. Amish Research Clinic of the University of Maryland, Baltimore is located in Lancaster, PA, USA, and nurses from this clinic recruited our study subjects. The inclusion criteria included: belonging to the Old Order Amish Community, being over 18 years old, and for our sub-project – having responded to a variant of a sleep questionnaire containing the variables of interest, as well as having *T. gondii* IgG titers and seropositivity results from a sub-study nested in the Amish Wellness Study that focused on the environmental and genetic risk factors for *T. gondii* infection. Informed consent for the parent Amish Wellness study was obtained after a thorough explanation of the study by nursing staff and Amish liaisons (Old Order Amish women working to secure a culturally sensitive interface between the Amish community and the nursing and medical staff of the University of Maryland Baltimore Amish Research Clinic). The protocol for the parent Amish Wellness study was approved by the University of Maryland, Baltimore Institutional Review Board.

The study sample comprised 833 Old Order Amish adults [mean age (SD)=44.28 (16.99) years], which included 341 (40.94%) men and 492 (59.06%) women. They each responded to sleep questionnaires as part of a wellness screen in Lancaster, PA. The questionnaires were vetted by the Amish liaisons and by nursing personnel familiar with the Amish participants and culture.

Fasting blood samples were centrifuged for 25 minutes at 400 *g* and at 4°C. Plasma was separated and stored at −80°C. To determine *T. gondii* serologic status, an enzyme-linked immunosorbent assay (ELISA) (IBL International, Männedorf, Switzerland) was used to test the plasma for IgG antibodies to *T. gondii*. The maximum dilution was 1:20. Status of each of the samples (i.e., seropositive or seronegative for infection with *T. gondii*) was defined by a predetermined cutoff value of 10 IU/mL, with a mean coefficient of variation of 7%, as reported by the ELISA manufacturer. When ELISA results indicated an equivocal concentration of *T. gondii* IgG (8 to 12 IU/ mL), the test was repeated. If the level was under 8 IU/ mL or over 12 IU/mL, it was considered seronegative or seropositive for infection with *T. gondii*, respectively. The individuals with persistent equivocal results were excluded from analysis.

#### Informed consent:

Informed consent was obtained from all individuals included in this study.

#### Ethical approval:

The research related to human use was in compliance with all the relevant national regulations, institutional policies and in accordance with the tenets of the Helsinki Declaration, and was approved by the authors’ institutional review board or equivalent committee.

### Sleep questionnaire

Selected questions were administered to measure our variables of interest, which were all analyzed in binary form. Sample sizes per variable differed due to the availability of *T. gondii* serology results in those subjects that had answered the sleep questionnaire. The questions “trouble staying asleep” (sleep maintenance), “problems during the day due to poor sleep” and “daytime sleepiness” were answered by 833 individuals, 455 (54.62%) of which were *T. gondii* seropositive. Other questions regarding sleep that did not include the ones mentioned above (because of a change in questionnaire length due to logistical issues), were previously analyzed in relationship to markers of chronic *T. gondii* infection [46]. Bedtime and wake-up time were self-reported and the mid-sleep time was calculated. All sleep timings were converted to decimal hours for analysis.

### Statistical methods

The relationship between *T. gondii* IgG seropositivity and transformed ranked answers from sleep questionnaires were analyzed using binary logistic regression, adjusted for age and sex (SAS version 9.3 SAS Institute Inc., Cary, NC, USA). The associations of *T. gondii* IgG titers with sleep duration and timing were analyzed using Linear Mixed Models adjusted for age, sex and family aggregation, using the Mixed Model Analysis for Pedigrees and Populations (MMAP) [55].

## Results

Digitally transformed timing of average bedtime (SD) was 21.38 (0.54), and average wake-up time (SD) was 05.14 (0.72). Average mid-sleep time (SD) was 01.26 (0.49). In analog clock-time our participants’ average bedtime was 9:22 PM and wake-up time was 5:08 AM.

### *T. gondii* seropositivity and sleep parameters

There were 455 (54.61%) *T. gondii* seropositive and 378 (45.39%) *T. gondii* seronegative participants. Sleep variables in the overall sample and stratified by *T. gondii* seropositivity status are presented in Table 1.

After adjustment for age and sex, *T. gondii* seropositivity had a significant negative association with difficulty staying asleep [OR 0.79, 95% CI: 0.68-0.92] and problems during the day due to poor sleep [OR 0.77, 95% CI: 0.64-0.92].

### *T. gondii* serointensity and sleep parameters

*T. gondii* serointensity was significantly negatively associated with bedtime and mid-sleep time, and positively associated with sleep duration. Specifically, higher *T. gondii* serointensity was associated with earlier bedtime (*β* = −0.04, *p* = 0.0002), earlier mid-sleep time (*β* = −0.002, *p* = 0.02) and longer sleep duration (*β* = 0.04, *p* = 0.02). Wake-up time (*β* = −0.0009, *p* = 0.07) was not significantly changed with *T. gondii* serointensity (trend towards statistical significance).

## Discussion

To our knowledge, the current study is the first to find a significant association between markers of *T. gondii* infection and sleep duration, timing and maintenance, as well as problems during the day due to poor sleep. The direction of these significant associations was, in part, surprising. Earlier, rather than later, bedtime and midsleep time, and longer rather than shorter sleep duration, were associated with higher *T. gondii* IgG titers. Moreover, *T. gondii* IgG seropositive individuals reported less, rather than more, difficulties in maintaining sleep and had fewer problems during the day due to poor sleep.

One avenue by which *T. gondii* may influence sleep is directly through its endogenous production of dopamine [53, 54, 56]. Evidence indicates that there is a relationship between dopamine and sleep-wake cycles. Patients with Parkinson’s disease, which reduces dopamine-producing neurons [57], often have excessive daytime sleepiness [58]. Nishino et al. (1998) demonstrated that dopamine-specific reuptake blockers increase wakefulness in normal and narcoleptic animals [59]. Wisor et al. (2001) reported increased wakefulness and reduction in NREM sleep in mice with the absence of the dopamine transporter gene [60]. A study done on dopamine (D_2_) receptor knockout mice showed decreased wakefulness, shorter wake periods, increased NREM sleep and increased stage transitions between being awake and NREM sleep [61]. Additionally in this study, sleep quality was also affected, as indicated by the lower delta activity, a component of deep sleep [61]. These data indicate the possibility that dopamine promotes wakefulness.

Chronic infection with *T. gondii* may also promote daytime wakefulness, as suggested by better self-reported tolerance of daytime consequences of poor sleep in our study sample. One way to explain the wakefulness effect is by dopamine’s implication in homeostatic and circadian components of sleep/wake regulation, which in turn are involved in numerous interactions with the neural, endocrine and immunological systems. Dopamine projects from the ventral tegmental area and substantia nigra, both of which contain functional clocks that schedule activities in a circadian manner [62-64], and leads to the promotion of wakefulness [58, 59, 61]. *T. gondii* can alter dopamine production in several ways. Firstly, it possesses tyrosine hydroxylase [54], the rate-limiting enzyme in dopamine production [65]. Secondly, inflammation secondary to *T. gondii* infection may activate the kynurenine pathway, leading to decreased brain levels of kynurenic acid, higher levels of which have been reported to have an inverse relationship with dopamine levels [66]. Thirdly, immune activation (necessary to contain *T. gondii* in immunocompetent hosts) may also interact with dopaminergic processes by inducing alterations in the level of tyrosine, the precursor of dopamine [65]. A high phenylalanine:tyrosine ratio, resulting from inhibition of phenylalanine hydroxylase [67, 68], can be the consequence of Th1 activation, one of the central immune mechanisms responsible for containing *T. gondii* infection in immunocompetent hosts [69]. We recently reported how associations between *T. gondii* and aggression [70] or impulsivity [71], known to be in part modulated by dopaminergic pathways, interact with plasma peripheral levels of phenylalanine:tyrosine ratios.

Independent of dopaminergic mechanisms, as any microbial organism, *T. gondii* can promote sleep through the induction of the immune system. Immune pathways that aid in the containment of *T. gondii*, as well as other infections in the immunocompetent host, have also been implicated in sleep-wake regulation. For instance, levels of IL-12 and TNF, both proinflammatory cytokines that have been linked to the acute control of *T. gondii* infection [72-75], peak during sleep regardless of circadian factors [1]. The activation of TNF initiates sleep [76]. IL-10, an anti-inflammatory cytokine, prevents overly active immune response during infection [77] and also peaks during sleep [1]. It is possible that low-grade immune activation, potentially as a direct consequence of *T. gondii* infection, is involved in mediating the relationship of *T. gondii* IgG seropositivity and IgG titers with sleep duration and reduced problems in maintaining sleep. This could be evaluated in future longitudinal studies with multiple immune markers.

The evolutionary pull of survival may, in part, be driving a parasite’s ability to protect sleep in its host thereby benefitting both host and pathogen. The host who sleeps longer with minimal nightly interruptions can have better health and may be more equipped to contain a pathogen through energy conservation [1, 3], ultimately reducing the chance of death, other than via predation by a representative of the cat family. Moreover, specifically for *T. gondii*, increased activity and reduced neophobia during wakefulness (as described in infected rodents) [78], may increase chances of rodent predation by a feline, thus completing the parasite’s life-cycle.

An alternative possibility is that some strains of *T. gondii* act as microbial “Old Friends” with immunoregulatory capabilities [79] and subsequently, provide their intermediate host with a mutually beneficial relationship, such as less problems related to sleep, healthier timing and duration of sleep, with a greater longevity of the host, as well as a longer exposure to predation by the members of the cat family, eventually, resulting in a greater chance for *T. gondii* to reproduce. This would not be an isolated clinical link as it is known, for example, that *T. gondii* infection is associated with lower incidence of allergy and asthma [80, 81], conditions that have also been linked to poor sleep [82, 83]. Although *T. gondii’s* common route of infection in humans is through the intestinal wall, there are relatively few studies focused on chronic toxoplasmosis and long term consequences on the gut microbiome, and further on immune function studies in human or nonhuman animals. In one small study, chronic *T. gondii*-infected mice did have an increase in gut immunomodulatory bacteria compared to uninfected mice [84].

### Strengths of the study

The Amish have a relatively homogeneous lifestyle [85] and have high rates of *T. gondii* seropositivity [86]. Limited alcohol and substance use consumption, as well as the absence of exposure to bright or blue artificial lighting from television sets, cell phones and computers [85] that alter the circadian rhythm and perturb sleep [87], are additional strengths of our study.

### Limitations

We had a cross-sectional design, did not adjust for multiple comparisons (with only the negative association between bedtime and *T. gondii* IgG serointensity being able to resist a full adjustment for multiple comparisons, post hoc), and used self-report sleep measures rather than objective measures. Since, the study was done in the Old Order Amish, the generalizability of the results is limited. We have not accounted for markers of inflammation and have not analyzed IgM and other markers to differentiate acute from chronic *T. gondii* infection. We cannot be sure if the lack of a significant association of titers with wake-up time is a result of more dominant environmental (likely occupational demands) factors, in comparison to midsleep and bedtime, or it being a consequence of a type II error due to a limited sample size, resulting in a statistical trend rather than achieving statistical significance.

In sum, *T. gondii* IgG seropositivity is associated with healthier sleep profiles, specifically having fewer problems maintaining sleep and fewer daytime problems due to poor sleep, and higher *T. gondii* IgG titers are associated with earlier bedtime and mid-sleep time, and longer sleep duration. Thus, there is no current evidence to support deleterious associations between *T. gondii* infection and sleep; hence, there are no grounds to continue to expect that sleep impairment could mediate the predictive associations between *T. gondii* infection and mental illness, suicidal behavior, or traffic accidents. Our results need to be replicated with longitudinal designs using objective measures of sleep. Future studies should also investigate potential immune mechanisms mediating these associations by measuring a comprehensive panel of inflammatory markers. For instance, given the high seroprevalence of *T. gondii* and considerable rate of seroconversion in the Amish, we can focus on measures of sleep continuity and timing on actigraphic tracing, or even polysomnography at baseline in *T. gondii* seronegative individuals, and then compare sleep patterns and differences from baseline in individuals who seroconverted versus those who remained seronegative. At several (at least two) time points, sleep questionnaires, sleep latency, wake-maintenance tests, sleep onset and offset and tests of alertness, such as the psychomotor vigilance tests [88], could be related to *T. gondii* seropositive status, serointensity, cytokine profiles and potential structural and functional neuroimaging. Additionally, circadian markers, such as dim light saliva melatonin onset [89, 90], could lead us closer to understanding the direction of causality and mechanisms of the unexpected associations revealed by our cross-sectional study.

## Figures and Tables

**Table 1:** Sleep parameters and *T. gondii* seropositive/seronegative status.

Sleep parameters	All	*T. gondii* (+)	*T. gondii* (−)
Trouble falling asleep (*n* = 829)			
No	593 (71.53%)	330 (39.81%)	263 (31.72)
Yes	236 (28.47%)	122 (14.72%)	114 (13.75%)
Trouble staying asleep (*n* = 833)			
No	415 (49.82%)	239 (28.69%)	176 (21.13%)
Yes	418 (50.18%)	216 (25.93%)	202 (24.25%)
Sleep quality (*n* = 828)			
Poor	13 (1.57%)	5 (0.60%)	8 (0.97%)
Good	815 (98.43%)	450 (54.35%)	365 (44.08%)
Problems during the day due to poor sleep (*n* = 833)			
No	668 (80.19%)	381 (45.74%)	287 (34.45%)
Yes	165 (19.81%)	74 (8.88%)	91 (10.92%)
Daytime sleepiness (*n* = 833)			
No	337 (40.46%)	199 (23.89%)	138 (16.57%)
Yes	496 (59.54%)	256 (30.73%)	240 (28.81%)

## List of abbrevations

ELISA: Enzyme-linked immunosorbent assay
GABA: Gamma-aminobutyric acid
IgG: Immunoglobulin G
IL: Interleukin
NREM: Non-rapid eye movement
T. gondii: Toxoplasma gondii
TNF: Tumor necrosis factor
